# Percutaneous transhepatic biliary drainage assisted by real-time virtual sonography: a retrospective study

**DOI:** 10.1186/1471-230X-13-127

**Published:** 2013-08-13

**Authors:** Masaya Miyazaki, Kei Shibuya, Hiroyuki Tokue, Yoshito Tsushima

**Affiliations:** 1 Department of Diagnostic and Interventional Radiology, Gunma University Hospital, 3-39-15 Showa-machi, Maebashi, Gunma 371-8511, Japan

**Keywords:** Ultrasound, Percutaneous transhepatic biliary drainage, Real-time virtual sonography, Magnetic navigation

## Abstract

**Background:**

Real-time virtual sonography (RVS) is a diagnostic imaging support system that can synchronize with ultrasound images in conjunction with computed tomography or magnetic resonance images using magnetic navigation system. RVS has been applied in clinical practice to perform such procedures as radiofrequency ablation and biopsy; however, the application of RVS for percutaneous transhepatic biliary drainage (PTBD) is rare.

**Methods:**

Between 2007 and 2012, RVS-assisted PTBD was performed for 30 patients (19 males and 11 females; age range, 41 to 89 years; mean age, 66.9 years) with obstructive jaundice. The targeted bile duct was determined using the RVS system before the procedure. The intervention was considered to be successful when the targeted bile duct was punctured and the drainage catheter was placed in the bile duct. Complications were evaluated according to the Society of Interventional Radiology Clinical Practice Guidelines.

**Results:**

A total of 37 interventions were performed for 30 patients. The interventions were successful in 35 (95%) of 37 interventions. The targeted bile ducts were: B3 (n = 24), B5 (n = 7), B8 (n = 3), B6 (n = 1), and the anterior (n = 1) and posterior (n = 1) branches of the right bile duct. The mean targeted bile duct diameter was 4.9 mm (1.9 to 8.2 mm). PTBD was able to be accomplished in all patients because the non-targeted bile ducts were successfully punctured alternatively. No major complications were observed in relation to the interventional procedure.

**Conclusions:**

RVS-assisted PTBD is a feasible and safe procedure. Accurate puncture of targeted bile ducts can be achieved using this method.

## Background

Percutaneous transhepatic biliary drainage (PTBD) is a common and effective procedure for the palliation of cholestasis in both malignant and benign biliary obstruction, especially after the application of the B-mode ultrasonography guidance [1-5]. Success rates for PTBD have been reported at 90% or more, and complication rates have been reported at 3% or less [6]. However, when applied to patients with a special condition (for example, a nondilated bile duct, patient status post left hepatic lobe resection, patient status post liver transplantation), PTBD has the potential for technical difficulties. In addition, for new practitioners of this procedure, understanding the bile duct anatomy (especially the right hepatic bile duct) can be challenging.

Real-time virtual sonography (RVS; Hitachi Medico, Tokyo, Japan) is a diagnostic imaging support system that can synchronize with B-mode ultrasound images in conjunction with two-dimensional multiplanar reconstruction (MPR) using a magnetic navigation system. RVS provides the same cross-sectional MPR images of the liver as ultrasound images on the same monitor screen in real time using volume data from multidetector computed tomography (MDCT) or magnetic resonance imaging (MRI) [7,8]. Recently, the RVS system has been applied in clinical practice to perform such procedures as radiofrequency ablation (RFA) for hepatocellular carcinoma (HCC) and biopsy for liver tumors [9-14]; however, the application of RVS for PTBD is rare.

In the present study, we evaluated the safety and efficacy of PTBD as assisted by the RVS system for the palliation of cholestasis.

## Methods

### Patients

Between December 2007 and July 2012, virtual sonography–guided PTBD was performed in 30 patients (19 males and 11 females; age range, 41 to 89 years; mean age, 66.9 years) with obstructive jaundice due to biliary obstruction. The causes of biliary obstruction were: bile duct carcinoma (n = 7), recurrent gastric carcinoma (n = 7), carcinoma of the pancreas (n = 5), gallbladder carcinoma (n = 3), bile duct stone (n = 2), biliary stenosis after liver transplantation (n = 2), hepatocellular carcinoma (n = 2), metastasis to the porta hepatis (n = 1), and primary peritoneal carcinoma (n = 1) (Table 1).

**Table 1:** Patient’s characteristics

**No.**	**Sex**	**Age**	**Characteristics of disease**	**Targeted bile duct**	**Diameter of the bile duct (mm)**	**Success of procedure**
1	M	89	Hilar bile duct Ca.	B3	8.2	Yes
2	M	69	Pancreas Head Ca.	B3	6.8	Yes
3	F	72	Pancreas Head Ca.	B3	3	No
				Ant. Branch	4.8	Yes
4	M	70	Pancreas Head Ca.	B3	5.3	Yes
5	M	55	Gastric Ca.	B3	3	Yes
6	M	75	Hepatocellular Ca.	B3	4.1	Yes
7	M	59	Hilar bile duct Ca.	B3	6.4	Yes
				B6	3.9	No
				B8	3.4	Yes
8	M	66	Gastric Ca.	B8	7.6	Yes
9	F	75	Gall bladder Ca.	B3	6.9	Yes
10	F	74	Bile duct Ca.	B3	5	Yes
11	M	73	Hilar bile duct Ca.	B8	7	Yes
12	F	48	Liver mets	B3	4.2	Yes
13	M	66	Pancreas Head Ca.	B3	4.2	Yes
14	M	66	Gall bladder Ca.	B3	4.5	Yes
15	F	51	Post Liver transplantation	B3	4.5	Yes
16	F	72	Panc. Body Ca.	B3	4.5	Yes
17	M	82	Bile duct Ca.	B5	5	Yes
18	M	68	Bile duct stone	B3	7.6	Yes
19	M	68	Gastric Ca.	B3	5.4	Yes
20	M	61	Bile duct stone	B3	3.5	Yes
21	M	61	Gastric Ca.	B3	5.3	Yes
22	F	68	Hilar bile duct Ca.	B3	4.2	Yes
23	M	72	Gall bladder Ca.	B3	6.8	Yes
24	F	60	Post Liver transplantation	B5	4.4	Yes
25	M	82	Gastric Ca.	B5	5.1	Yes
				Post. Branch	5.2	Yes

The study was conducted in accordance with the principles of the Helsinki Declaration. It was approved by the ethics committee of the Gunma University Hospital (12-48).

### Real-time virtual sonography

**Figure 1:**
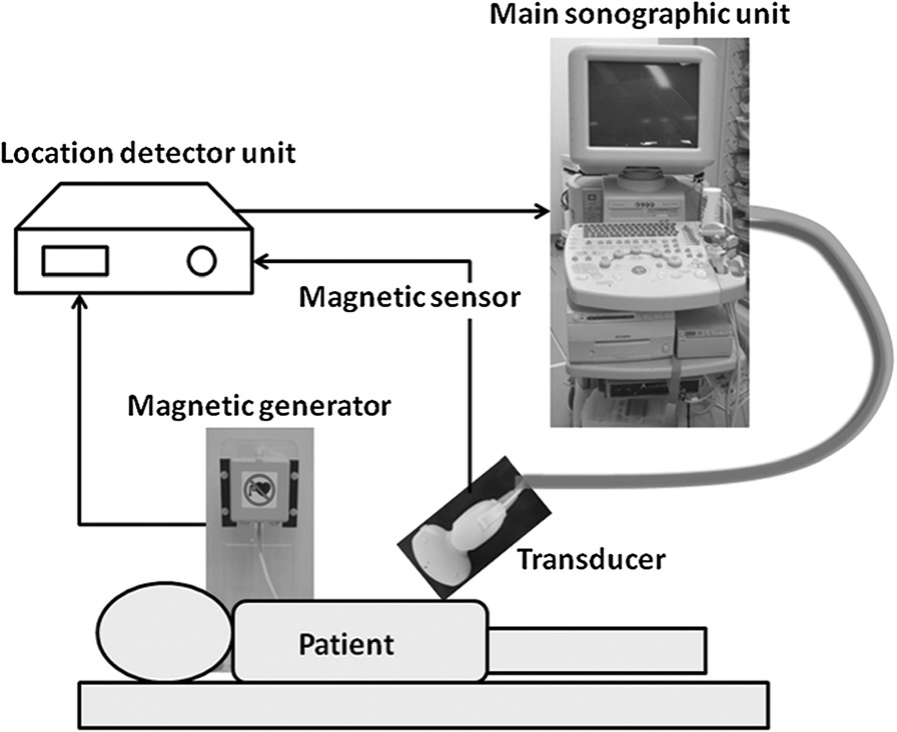
**The RVS system consists of a main unit, a magnetic location detector unit, a magnetic field generator and a magnetic sensor.** The sensor precisely and consistently captures changes in magnetic fields produced by the generator and detects changes in the location, direction, and rotation of the transducer scanning the patient. The detector instantaneously processes changes in positional information detected by the magnetic sensor and transfers this information to the main unit. The RVS main unit generates and displays real-time MPR images matching cross-sectional images of the abdomen captured by the transducer. RVS: Real-time virtual sonography; MPR: multiplanar reconstruction.

The ultrasound device used was an RVS system (EUB 7500, Hitachi Medical Corporation, Tokyo, Japan). The RVS system consists of a main ultrasonographic unit, a magnetic location detector unit, a magnetic field generator, and a magnetic sensor. The magnetic field generator is fixed to the left side of the fluoroscopy bed, and the magnetic sensor is attached to the sonographic transducer. The magnetic sensor precisely and consistently captures changes in magnetic fields produced by the generator and detects changes in the location, direction, and rotation of the transducer scanning the patient using the detector unit. The detector instantaneously processes changes in positional information detected by the magnetic sensor and transfers this information to the main unit. The RVS main unit generates and displays real-time MPR images matching cross-sectional images of the abdomen captured by the transducer (Figure 1).

The actual examination is as follows: 1) Before the procedure, the Digital Imaging and Communication in Medicine (DICOM) volume data from computed tomography (CT) or magnetic resonance imaging (MRI) is loaded into the main unit. 2) After the RVS software is started, a CT/MRI image is selected that was obtained at the caudal level of the xiphoid and placed at the center of the transducer attached to a magnetic sensor lengthwise on the skin at the caudal level of the xiphoid. 3) The xiphoid process serves as the initial reference point. When a CT/MRI image with the tip of the xiphoid process captured before MPR processing matches the tip of the xiphoid process seen on the actual abdominal ultrasound image, the RVS system provides the same virtual MPR image corresponding to the movement of the transducer on the same monitor screen in real time. 4) B-mode ultrasound images are synchronized with the virtual MPR image at the best timing of the patient’s breathing. When the images show a gap between the virtual MPR image and B-mode sonography, the view is adjusted. The view of the umbilical portion or of the right portal vein is searched on the virtual MPR image using the sonographic transducer. After the view is fixed, the virtual MPR image is released with the corresponding B-mode sonographic image. 5) Finally, synchronous images from virtual CT sonography and B-mode sonography are obtained side-by-side on the monitor screen of the main unit.

**Figure 2:**
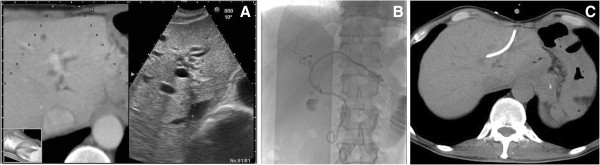
**A 55-year-old man with jaundice due to peritonitis carcinomatosa from gastric cancer. A**: The virtual CT sonographic image (left) displayed with the corresponding B-mode sonogram (right) shows the dilated bile duct (B3: arrow). The virtual image shows that the stomach cavity (arrow head) is close to the lateral segment of the liver. However, a B-mode sonogram cannot show the stomach cavity in the same view. The virtual image helps to determine the safe puncture route. **B**: The radiograph shows successful internal-external bile duct drainage using a 8.5 French drainage catheter. **C**: The CT image shows that the drainage catheter is inserted into the bile duct through the route planned before the procedure using the RVS system. CT: computed tomography.

**Figure 3:**
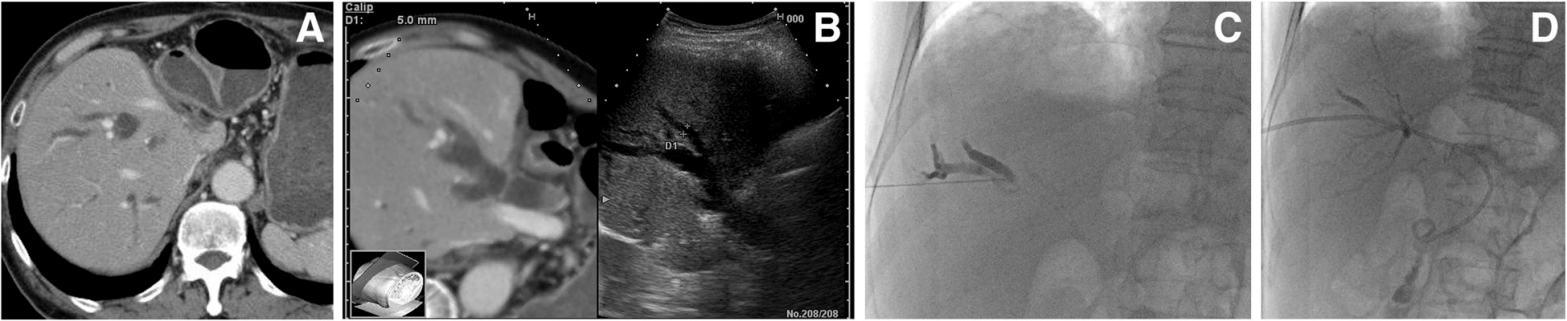
**An 82-year-old man with jaundice due to lymphadenopathy of the porta hepatis from a sigmoid cancer. A**: The CT image shows a dilated bile duct in the right liver lobe. The stomach cavity is in contact with the liver because the lateral hepatic segment was resected due to a liver metastasis. **B**: The virtual CT image and the B-mode sonogram show the dilated bile duct (B5: arrow). The virtual image shows that the stomach cavity (arrow head) is close to the liver; a B-mode sonogram cannot show this. **C**: The radiograph shows that the needle is safety inserted into the bile duct, avoiding the stomach as guided by the RVS system. **D**: The radiograph shows successful internal-external bile duct drainage.

Before the PTBD procedure, the anatomical relationships between the bile duct and the gastrointestinal tract or intrahepatic vessels are examined and an appropriate puncture route to the bile duct is searched on the RVS system. After the examination, the targeted bile duct is determined (Figures 2 and 3).

### Standardized PTBD protocol

The patients were premedicated with an analgesic (fractionated IV injection of a maximum of 15 mg of pentazocine hydrochloride [Pentagin; Daiichi-Sankyo, Tokyo, Japan], followed by skin disinfection and local anesthesia (10 mL of 1% lidocaine [Xylocaine® injection; AstraZeneca, Osaka, Japan]). Under B-mode ultrasonographic guidance, a 21-gauge Chiba needle (Top, Tokyo, Japan) was inserted into the targeted bile duct that had been determined on the RVS system before the procedure. If puncturing the targeted bile duct proved difficult despite several attempts, puncturing of the other bile duct was allowed and the change in protocol was recorded. Upon successful placement of the needle tip in a bile duct, a 0.018-inch guidewire (PTCD-K-2; Cook Japan, Tokyo, Japan) was advanced, followed by insertion of a coaxial dilatation catheter (PTCD-K-2; Cook Japan, Inc.) using the Seldinger technique. Next, the initial thin-lumen guidewire was exchanged for a 0.035-inch Cook guidewire (Cook Japan, Inc.). The Cook guidewire served to probe the common bile duct and was then advanced through the papilla into the small intestine, if possible. After removal of the introducer sheath, the final PTBD catheter (8.5 French Ultrathane® drainage catheter set, Cook Japan, Inc.), with multiple side holes positioned on both sides for internal-external drainage, was then advanced over the wire. If the guidewire was difficult to advance into the common bile duct or the small intestine, the tip of the drainage catheter was placed in the peripheral bile duct. In addition, internal-external drainage was not performed in cases when a surgical resection had been already planned for treating jaundice in order to avoid unexpected tumor bleeding and other complications.

### Evaluation of the procedure

We performed a radiological review and medical record review of all the study patients, noting the success rates of the procedures and occurrence of complications. The intervention was considered to be successful when the targeted bile duct was punctured and the drainage catheter was placed into the bile duct. The success of puncturing the targeted bile duct was judged by CT or MRI images that were performed after the procedure. In addition, the puncture site of the bile duct and the diameter of the bile duct were also recorded. Complications were evaluated according to the Society of Interventional Radiology (SIR) Clinical Practice Guidelines [15].

## Results

A total of 37 bile duct interventions were performed in 30 patients (Table 1). The interventions were successful in 35 (95%) of the 37 procedures. Internal-external drainage was attempted in 30 interventions, and was successful in 19 (63%) interventions. The targeted bile ducts were: B3 (n = 24), B5 (n = 7), B8 (n = 3), B6 (n = 1), and the anterior (n = 1) and posterior (n = 1) branches of the right bile duct. Two targeted bile ducts (B3 and B6) failed to be punctured in the first intervention; however, other bile ducts were punctured alternatively and the drainage catheter was placed in a bile duct. Thus, bile drainage was accomplished in all 30 patients. The mean targeted bile duct diameter was 4.9 mm (1.9 to 8.2 mm). The reference images for the RVS system were: contrast-enhanced CT images (n = 31), plain CT images (n = 4), contrast-enhanced T1-weighted MR images (n = 1), and combined contrast-enhanced CT and T2-weighted MR images (n = 1). No major complications (SIR Classification C–F) related to the interventional procedure was observed in any of the patients. There were 16 (53%) minor complications (SIR Classification A or B) observed that were related to the procedure, including slight hemobilia, temporary fever, abdominal pain, puncture route pain, and nausea.

## Discussion

Percutaneous transhepatic biliary drainage is a therapeutic procedure for patients with cholestasis, whether from malignant or benign biliary obstruction. PTBD is a widely accepted procedure because a greater than 90% success rate has been reported in several studies [6]. However, PTBD for a patient with a nondilated bile duct is sometimes difficult, with the success rate varying from 50% to 100% [4,5,16]. Therefore, new techniques and devices are required for performing advanced PTBD procedures.

RVS has recently been introduced as a new technique for the performance of some interventional procedures, such as liver biopsy and RFA. Some authors have reported the efficacy of RFA for HCC using RVS [9-14]. To the best of our knowledge, the application of RVS to PTBD has not yet been reported in the literature. In the present study, all PTBD procedures were performed with RVS without major complications. RVS showed the anatomical relationships between the bile duct and the gastrointestinal tract or intrahepatic vessels before puncture. The targeted bile ducts were successfully punctured in 95% of the study subjects, and bile drainage was accomplished in all subjects. Precise puncture to the peripheral bile duct is important to several aspects of PTBD, such as insertion of the metallic stent into the obstructed bile duct. RVS is needed for the safe puncture of the peripheral bile duct, because the clear visualization provided by RVS helps avoid riskier puncture routes that include the gastrointestinal tract or major vessels.

Puncture of right bile duct is more difficult than puncture of the left bile duct because visualizing and understanding the three-dimensional biliary anatomy of the right liver lobe is difficult under B-mode ultrasonographic guidance [17,18]. RVS can display the image corresponding to the movement of the transducer in the magnetic field, allowing the practitioner to see synchronous images from the virtual sonographic image and B-mode sonography side-by-side on the sonographic monitor. Therefore, RVS facilitates easier puncture of the right bile duct because the targeted bile duct can be easily determined with this system. Although there were only nine punctures to the right bile duct in the present study, all interventions except one were successful in puncturing the targeted bile duct.

In the present study, enhanced CT was commonly used as a reference image for RVS. However, MRI can be used as a reference image for RVS if formatted using the DICOM standard. RVS with MRI imaging might be available for patients who are allergic to CT contrast medium. In addition, T2-weighted MR images without contrast medium might be more suitable for reference imaging when RVS is used for a patient with jaundice, because the bile duct is usually visualized clearly in T2-weighted MR images.

This study had several limitations. First, the number of cases was small, and the study design was retrospective. Second, the targeted bile ducts of this study were relatively dilated, and the success rate of PTBD for a dilated bile duct had already been quite high in previous studies [5,6,16]. Third, this study was not organized as the comparative study between PTBD using RVS and the conventional technique. Therefore, we cannot conclude that the success rate of PTBD using RVS is better than the conventional procedure. Further prospective comparative studies between this technique and the conventional technique in a large number of patients with nondilated bile ducts are needed.

## Conclusion

We have demonstrated the performance of PTBD using the RVS system as a novel technique. Accurate punctures of the targeted bile ducts were achieved in our study because RVS allowed visualization of the three-dimensional relationships between the bile ducts and surrounding tissues.

## Competing interests

The authors declare they have no competing interests.

## Authors’ contributions

MM was involved in the study design, data collection, data analyses, data interpretation, literature review, manuscript preparation, editing and submission. HT and KS were involved in the clinical care. YT was involved in the manuscript editing and review. MM will act as guarantor for the manuscript and is the corresponding author. All authors have read and approved the final manuscript.

## Pre-publication history

The pre-publication history for this paper can be accessed here:

http://www.biomedcentral.com/1471-230X/13/127/prepub
